# Supplementary material for: Individual differences in working memory impact the trajectory of non-native speech category learning
Source: PLoS One. 2024 Jun 10;19(6):e0297917. doi: 10.1371/journal.pone.0297917 (PMC11164376; doi:10.1371/journal.pone.0297917)
Supplement: S1 File — (DOCX) [file pone.0297917.s001.docx]

**Supporting Information**

**Figure A**

*Working memory and category learning performance considering attrition*


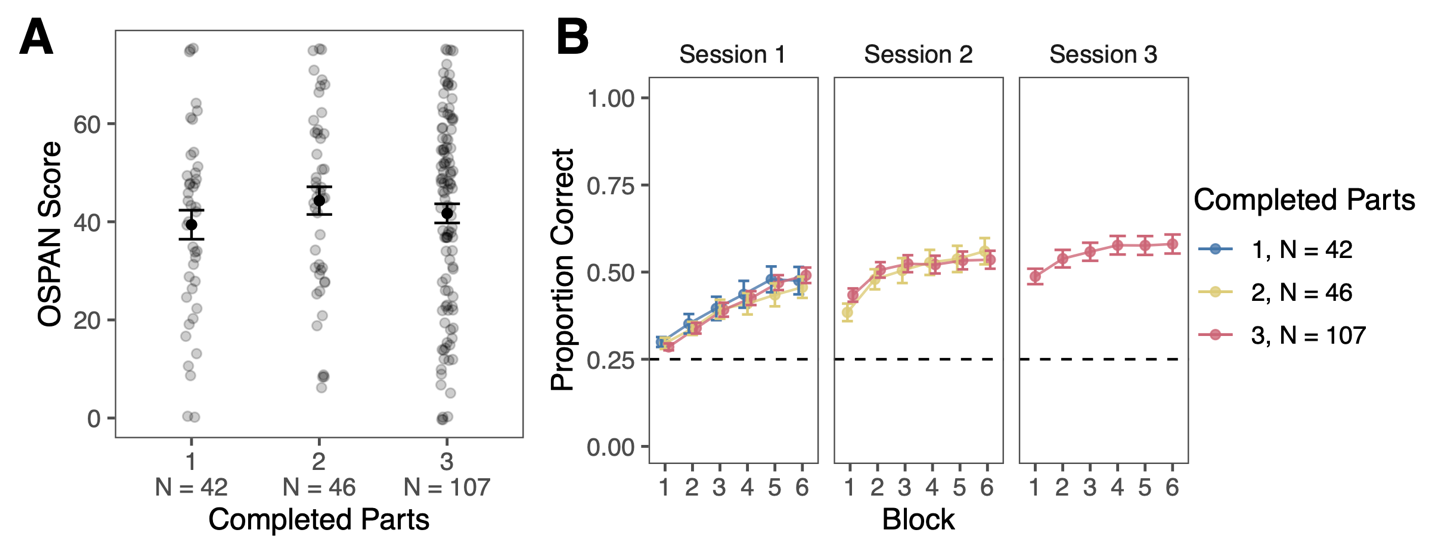


*Note.* Error bars reflect *SEM*.

Across participants who only finished a single session (*N* = 42), two sessions (*N =* 46) or all three sessions (*N* = 107), there were no significant differences in WM score (Figure A-a, *F*(2, 192) = 0.68, *p* = .51, η_G_^2^ = 0.007) or performance in the category learning task in session 1 or 2 (Figure A-b, Session 1: *F*(2, 192) = 0.15, *p* = .87, η_G_^2^ = 0.002; Session 2: *t*(95.9) = 0.39, *p* = .70, *d* = 0.067, 95% CI [-6.09, 9.06]). This indicates that attrition was not related to performance in the experimental tasks.

**Figure B**

*Accuracy on arithmetic questions in working memory task for learners and non-learners*

**
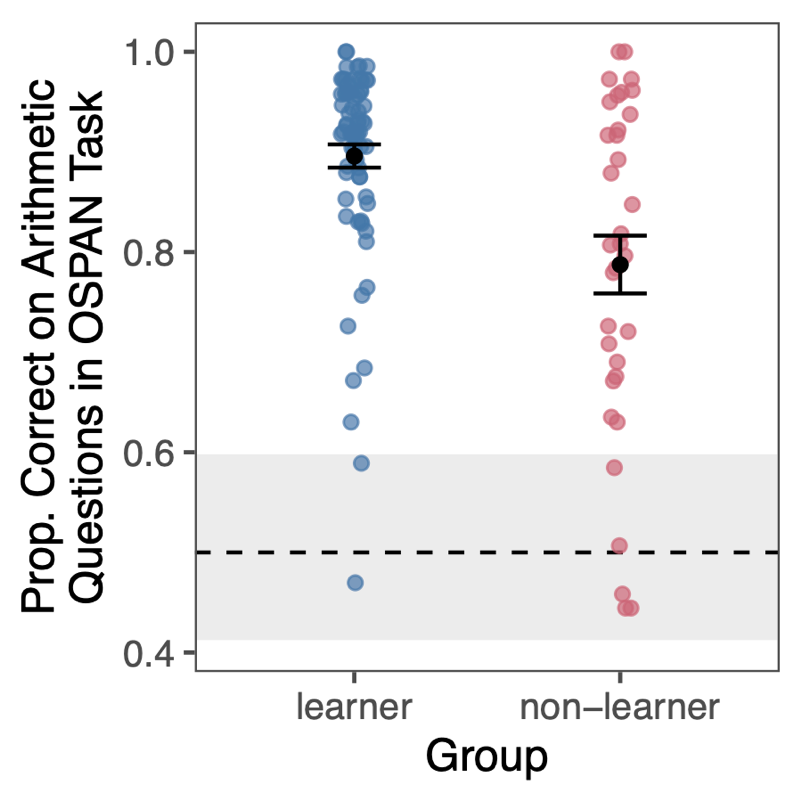
**

*Note.* Error bars reflect SEM, the dashed line reflects chance performance with the shaded region reflecting the 95% cumulative probability across 75 trials, 0.5 probability of success on each trial (50% +/- 8.67% chance).

Non-learners individuals (*M* = 79%) performed significantly worse on the correct/incorrect arithmetic questions in the OSPAN task compared to engaged individuals (Figure B; *M* = 90%; *t*(44) = 3.49, *p* = .0011, 95% CI [4.58, 17.1]). This indicates that non-learners may have been generally disengaged in the experiment.

**Overview of the Drift-Diffusion Model**

We have used here a drift-diffusion model adapted from the recent work by Paulon et al. (2021). The full details of the drift-diffusion model, including the choice of the priors, the resulting posterior, associated computational machinery for model fitting and posterior inference, simulated and real data illustrations, etc. can be found in Paulon et al. (2021).

In each trial, given an input stimulus $s$, the model assumes the evidence for different response categories $d$ to be accumulated via latent Weiner diffusion processes, one for each response category $d$ with respective drifts $\mu_{d,s}$, racing toward their respective boundaries $b_{d}$. The process reaching its boundary first corresponds to the response decision taken in the end and the time taken to reach this boundary corresponds to the associated response time. The model also included an offset parameter $\delta_{s}$ for each stimulus category, characterizing the times taken by the actions that are not directly relevant to the actual decision-making processes. The model allows individual heterogeneity in all parameters and dynamic variation in the drifts and the boundaries as ${\delta_{s}^{(i)}, \mu}_{d,s}^{\left( i \right)}\left( t \right), and b_{d}^{\left( i \right)}\left( t \right)$, where $i$ stands for the $i^{\mathrm{th}}$individual and $t$ for the $t^{\mathrm{th}}$training block. These time varying individual specific parameters $\mu_{d,s}^{\left( i \right)}\left( t \right)$ are modeled using an exponentiated additive model with two component functions as $\mu_{d,s}^{\left( i \right)}\left( t \right)= exp\{f_{\mu, d,s}\left( t \right)+ u_{\mu, d,s}^{\left( i \right)}\left( t \right)\}$, where $f_{\mu,d,s}\left( t \right)$ characterizes the fixed effects associated with the response-input combination, and $u_{\mu,d,s}^{\left( i \right)}\left( t \right)= u_{\mu,C}^{\left( i \right)}\left( t \right)$ when $d=s$ and $u_{\mu, d,s}^{\left( i \right)}\left( t \right)= u_{\mu,I}^{\left( i \right)}\left( t \right)$ when $d\neq s$ characterize the random effects associated with individual $i$ for correct (C) and incorrect (I) decisions respectively. Similarly, $b_{d}^{\left( i \right)}\left( t \right)= exp\{f_{b, d}\left( t \right)+ u_{b, d}^{\left( i \right)}\left( t \right)\}$, etc. These time-varying functions are all modeled using flexible smoothed mixtures of B-spline bases, the smoothness induced by Markovian priors that penalize large differences between adjacent time points. A uniform prior is assigned on the offsets $\delta_{s}^{(i)}$. Inference is based on samples drawn from the posterior via a Markov chain Monte Carlo (MCMC) algorithm. Full details of these priors and the algorithm can be found in Paulon et al. (2021).

We compared with a sub-model that allows the boundaries to still vary between $d$ and $i$ but assumes them to stay constant over time at $b_{d}^{(i)}$. We used Watanabe–Akaike information criterion (WAIC) to compare model fits (Zorzetto et al., 2023). Specifically, Table A reports negative 2*WAIC, a smaller value indicating better model fit. Based on their estimated values, the more flexible model with $b_{d}^{(i)}(t)$ provides significantly better model fit. We have also used predictive checks for a visual assessment of the model fit (Figure C). The more flexible model seems to match the data better.

**Table A**

Model fit (-2*WAIC) for different sub-models considered.

|  | Session 1 | Session 2 | Session 3 |
| --- | --- | --- | --- |
| Model with $b_{d}^{(i)}(t)$ | 93409.11 | 83795.52 | 77647.32 |
| Model with $b_{d}^{(i)}$ | 95697.74 | 85671.44 | 79635.96 |

**Figure C**

*Model fit based on predicted response time and accuracy by response category*

*
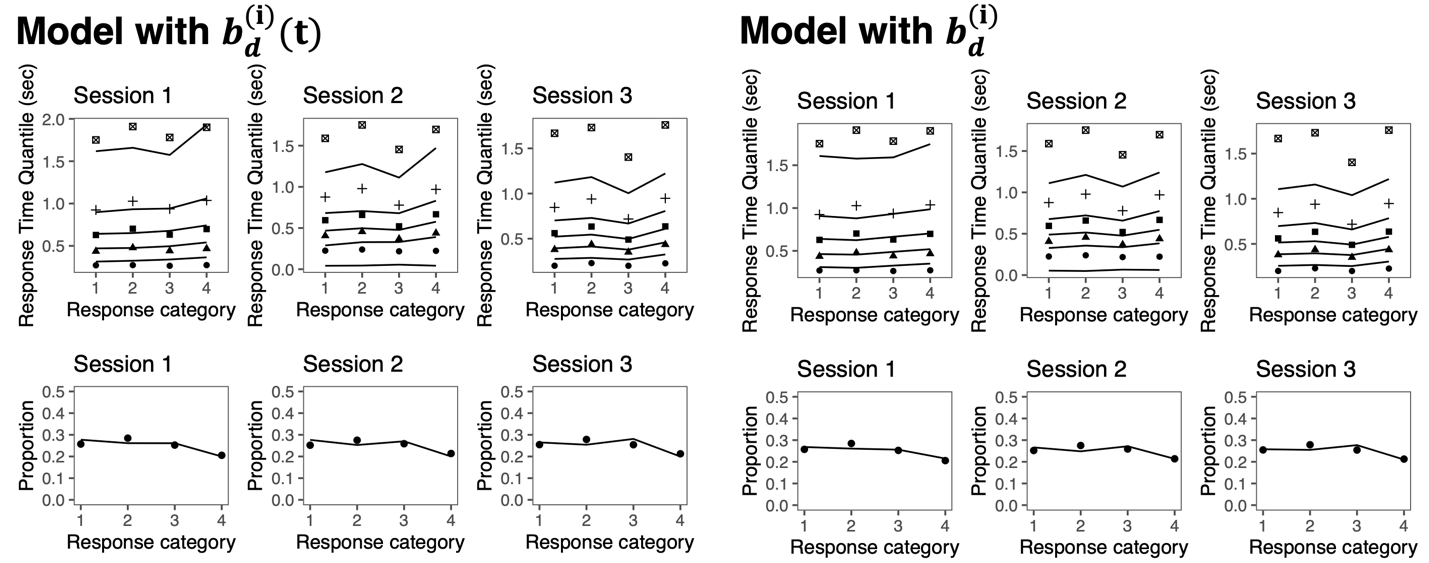
*

*Note.* Points reflect actual data, lines reflect model.

**Table B**

*Regression results for maintenance of learners’ category knowledge*

| Fixed Effect | Estimate | SE | *t* | *p* |
| --- | --- | --- | --- | --- |
| Intercept | 5.22 | 3.54 | 1.47 | .14 |
| Session 2 to 3 | -0.48 | 4.92 | -0.098 | .92 |
| No Delay | -4.67 | 4.92 | -0.95 | .34 |
| OSPAN | 0.035 | 0.072 | 0.48 | .63 |
| Session 2 to 3 * No Delay | 4.35 | 6.96 | 0.63 | .53 |
| Session 2 to 3 * OSPAN | 0.012 | 0.10 | 0.12 | .90 |
| No Delay * OSPAN | -0.18 | 0.10 | -0.18 | .86 |
| Session 2 to 3 * No Delay * OSPAN | -0.11 | 0.14 | -0.80 | .42 |

**Table C**

*Regression results for learners’ generalization*

| Fixed Effect | Estimate | SE | *t* | *p* |
| --- | --- | --- | --- | --- |
| Intercept | 43.0 | 5.89 | 7.30 | < .0001 |
| Session 2 | 14.7 | 4.04 | 3.63 | .00039 |
| Session 3 | 19.2 | 4.04 | 4.77 | < .0001 |
| OSPAN | 0.34 | 0.12 | 2.83 | .0056 |
| Session 2 * OSPAN | -0.14 | 0.083 | -1.62 | .11 |
| Session 3 * OSPAN | -0.18 | 0.083 | -2.13 | .035 |

**Figure D**

**
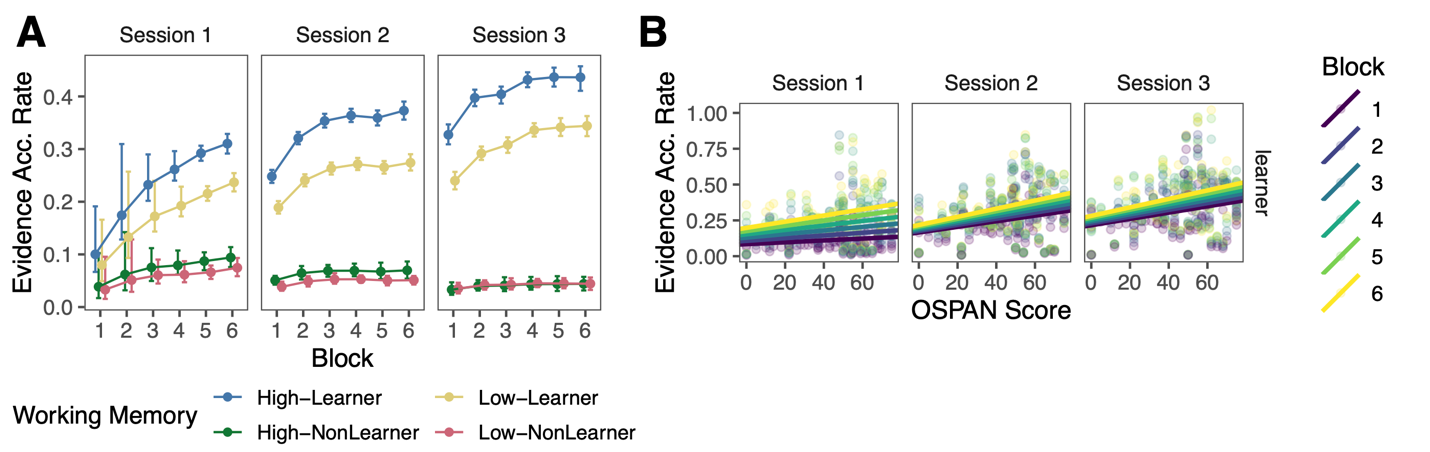
**

**Table D**

*Regression results for relationship between WM capacity and evidence accumulation rate for all responses*

|  | β | *SE* | *p* |
| --- | --- | --- | --- |
| Intercept | 0.061 | 0.043 | .16 |
| OSPAN | 0.00035 | 0.00089 | .70 |
| Block | 0.023 | 0.0053 | < .0001 |
| Session 2 | 0.094 | 0.029 | .0014 |
| Session 3 | 0.14 | 0.029 | < .0001 |
| OSPAN * Block | 0.00030 | 0.00011 | .0061 |
| OSPAN * Session 2 | 0.0014 | 0.00060 | .018 |
| OSPAN * Session 3 | 0.0017 | 0.00060 | .0050 |
| Block * Session 2 | -0.012 | 0.0075 | .10 |
| Block * Session 3 | -0.0097 | 0.0075 | .20 |
| OSPAN * Block * Session 2 | -0.00012 | 0.00016 | .43 |
| OSPAN * Block * Session 3 | -0.00015 | 0.00016 | .34 |

*Note.* Regression results are not given for decision threshold for all responses because this is reported in the main text.

**References**

Zorzetto, E., Canale, A., & Marani, M. (2023). A Bayesian non-asymptotic extreme value model for daily rainfall data. *Journal of Hydrology*, 130378. https://doi.org/10.1016/j.jhydrol.2023.130378
