# Supplementary material for: Individual differences in working memory impact the trajectory of non-native speech category learning
Source: PLoS One. 2024 Jun 10;19(6):e0297917. doi: 10.1371/journal.pone.0297917 (PMC11164376; doi:10.1371/journal.pone.0297917)
Supplement: Attachment — Submitted filename: 2023-04-25_ResponsetoReviewers.pdf [file pone.0297917.s002.pdf]

We thank the editor and three reviewers for consideration of our manuscript. Below, we briefly summarize the major changes we have made to the manuscript in response to reviewer comments. We then respond to each point raised by the reviewers with our responses in blue italics. We believe these changes significantly strengthen the manuscript.

Summary:

1. ***Drift diffusion model details and analyses.*** *To address comments from all three reviewers (R1: C2, 3, 11, 12, 15, 16, R2: C4, R3: C2, 3, 7, 8, 9), we have provided more information about the drift diffusion model parameters and analyses. We have also provided justification of our choice to allow boundaries to differ across stimulus and response categories and included a direct comparison of this full model with a sub-model that only allowed boundaries to differ across response categories. We demonstrated that the full model is psychologically plausible and provides a better statistical fit to the data than the sub-model. We believe these revisions substantially improve the manuscript and better contextualize the results.*
2. ***Block as a continuous/categorical variable.*** *Reviewer 2 (C1-3) raised valid concerns about our application of the regression models with block as a categorical variable. We agree with these concerns and have now re-ran the models using block as a continuous variable within sessions. We also directly compare performance across sessions. This enables us to better compare across blocks and sessions without an overly inflated alpha.*
3. ***Takeaway messages.*** *Reviewer 1 (C4) suggested that we edit the manuscript to make the takeaway messages clearer for readers. We have now drawn readers' attention to the main takeaways throughout the manuscript and believe that this strengthens the manuscript as a whole.*

Reviewer comments and responses

Reviewer #1

Roark et al. present the results of research aimed at assessing the role of working memory in the learning and retention of categorical knowledge about non-native language stimuli. Participants completed a task online in which they learned Mandarin tone categories across three sessions, separated by months. The authors assessed the relationships between various aspects of task performance and participants' performance on an operation span task. In particular, they focused on relationships between OSPAN task performance and initial and later learning, maintenance of category knowledge across sessions, generalisation between trained and non-trained stimuli, and drift rate and threshold parameters from a multi-alternative diffusion model used to fit the data. Results showed positive relationships between OSPAN scores and categorisation accuracy in later learning (e.g., later blocks of session 1 and most blocks of subsequent sessions); however, when the sample was split into learners and non-learners, this relationship was not evident in either group. Maintenance of category knowledge across sessions and across the final blocks within a session was also unrelated to OSPAN scores, as was generalisation from training to test stimuli. Diffusion modelling showed positive relationships between OSPAN scores and both drift rate and threshold in later blocks of session 1, and in subsequent sessions, for learners. The authors conclude that greater WM capacity may aid category learning in language contexts, particularly in the early stages of acquisition; and that higher WM capacity allows for greater caution in decision-making owing owing to more rapid evidence accumulation.

I should mention at the outset that whereas my knowledge of the WM literature and evidence accumulation models of decision-making is fairly good, I am far from an expert when it comes to research into language learning. My comments thus largely focus on these former aspects of the work. I should also note that this makes it difficult for me to judge the theoretical value of the contribution this research makes to the language learning area.

I have four major comments concerning the paper (not presented in any particular order), and a few more minor ones.

Major comments:
R1.C1: 1. My understanding is that theories of performance on complex span tasks (such as the OSPAN task) often assume a contribution from retrieval from LTM/episodic memory (e.g., Unsworth & Engle, 2007). If so, then I think a relevant question is, to what extent do the relationships between OSPAN performance and category learning shown here reflect the influence of LTM retrieval in both tasks? In other words, are the relationships evident in this work a result of the fact that both tasks to some extent tap how well people can retrieve stuff from LTM? Or is there something more to it than that? The meaning of the relationship depends on the answer to this question, and therefore I think it's something that would be worth addressing.
Thank you for raising this point. We now directly address the potential involvement of LTM/episodic memory to performance in the OSPAN task in the interpretation of our results in the Discussion on pages 37-38 (lines 796-806): "Further, because of the nature of the complex span task we used to assess WM capacity, it is possible that performance was influenced by some combination of WM and long-term memory [88]. As a result, the observed relationship between WM score and speech category learning performance may reflect the ability to hold onto and manipulate information in WM *as well as* retrieve exemplars or rules from long-term memory. However, it is important to note that measures that should theoretically be related to long-term memory or activation of exemplars stored in memory (e.g., maintenance, generalization) were not significantly related to WM score. Future studies should collect multiple measures of WM including visuospatial and auditory WM as well as measures of long-term memory to better understand how speech category learning relies on WM and long-term memory abilities."

R1.C2: 2. I was fairly confused by some aspects of the way the diffusion modelling was used in the paper. I had not come across this particular model before, but---if I understand it correctly---the basic idea seems straightforward: Assume Wald distributions for each response with densities that are multiplied by the product of the survival functions for the other responses. Nonetheless, there were two points that confused me:

a) At one point (p. 24) you mention focusing on "correct trials only". I followed the reference used here (Roark et al., 2021, PB&R) to try to make sense of this, and unfortunately became even more confused. The source of my confusion is this: I don't understand how you can isolate a drift rate and threshold for correct trials only when all the responses must surely influence the estimated parameter values. The only thing I could think of was that you were only fitting the data from trials with correct responses; but this would lead to heavily biased parameter estimates (with the bias stronger when the proportion of errors increases). As such, I think you either need

to clarify what it was you actually did, so that other readers don't end up as confused as I am; and/or re-do the modelling to fit all the data, rather than just those from correct trials. (On about my fifth read through the section, I think what you were actually doing here was fitting the model to all the data, but just reporting the parameters associated with the accumulator corresponding to the correct response. Right? This is more sensible than what I initially thought you were doing, but if this is correct, you really ought to clarify it so other readers don't go down the same garden path I did.)
We indeed fit the model to data from all trials comprising both correct AND incorrect responses to estimate the parameters. However, since gradual improvements in making correct decisions characterize learning, in our discussion of the results, we emphasized heavily on inferring the parameters associated with successful identification of the input stimulus. We have edited the Methods section (page 16, lines 340-344) to explain these points more clearly: "The remaining data, comprising both correct and incorrect trials, were used to estimate the parameters. Since gradual improvements in making correct decisions characterize learning, in our discussions below, we emphasize heavily on inferring the parameters associated with successful identification of the stimulus, that is, $\mu_{d,s}$ and $b_{d,s}$ for correct responses with $s = d$."

b) It seems to me that you use the estimated parameters for individual participants, obtained from the diffusion modelling, as the outcome variable in a regular linear regression with OSPAN score as the predictor. As I understand it though, the hierarchical structure of the diffusion model means these participant-level parameters are not independent, and therefore are not appropriate for use in the subsequent regression. Perhaps a better tack would be to include OSPAN scores as a predictor in the diffusion model itself.
We used the estimated parameters for individual parameters from the diffusion modeling as the outcome variable. A single-stage estimation method with the OSPAN scores as a predictor in the diffusion model would be statistically more appropriate, we agree. However, while the multi-category multi-subject time-varying longitudinal drift-diffusion mixed models we have used here is a very sophisticated one, it is not straightforward to incorporate external continuous covariates in this model - we would need to carefully design such models, develop associated computational machinery, rigorously test them, etc. This is beyond the scope of the current article, but we do plan to pursue this statistics methodology problem as the topic of a separate project. We also note that multi-stage methods are quite widely used in the scientific literature when single-stage methods are not available. Based on our experience (in other contexts), multi-stage models, especially when they involve many latent variables (as with latent diffusion processes in drift-diffusion models), often produce more efficient and numerically stable inference. Finally, for this particular problem, we do not think a single-stage implementation will change the scientific conclusions we arrived at using our two-stage procedure.

R1.C3: 3. Related to the previous point: If what you were doing with the diffusion modelling was indeed fitting all the data but just reporting parameters for the accumulator associated with the correct responses, I'd recommend also reporting the parameters for the other accumulators, at least in supplemental materials. In particular, when it comes to the effects of WM on thresholds (e.g., increasing with practice for higher-WM individuals), it's important to know whether this is unique to the accumulator for the correct response or consistent across all accumulators. If the effect is only evident on the accumulator for the correct response, this may indicate the model is not providing a good account of the data (since it is psychologically implausible that a

participant could selectively adjust the threshold on the accumulator for the correct response before they know it to be the correct response). The solution to this would be to fix the accumulators to have the same thresholds.
We now report the parameters for all accumulators in the Supporting Information – specifically, we present the relationship between OSPAN score and evidence accumulation rate/decision threshold for the average of all stimuli and responses rather than just correct responses. We note that this does not change the overall pattern of results.

R1.C4: 4. I found it a little difficult to get a clear picture of the primary message you're intending the reader to take from the research. As I mentioned above, I am not very familiar with the language learning literature, so this may be an artefact of that unfamiliarity (e.g., findings with an importance that would be obvious to someone actively working in the area were not obvious to me). Even so, if you want the work to be more accessible to a general audience, it might be worth thinking about how you can better emphasise this message.
We thank the reviewer for this comment. We have now better summarized the primary takeaway messages for readers in the Abstract, Introduction, and Discussion.

Minor comments:
R1.C5: p. 3, line 53: I think "temporal storage" should be "temporary storage".
We have changed this to "temporary storage"

R1.C6: pp. 3--4, lines 67--68: Really pedantic point here, but I think it's more precise to characterise performance as improving rather than increasing.
We have changed this to "improving"

R1.C7: p. 4: In the discussion of the role of WM capacity in language learning (or elsewhere), I wonder whether it might be worthwhile bringing in recent work by Smalle (e.g., Smalle et al., 2021, 2022; see references below) suggesting that interfering with cognitive control mechanisms (an important part of WM) can improve statistical learning (an important part of language development)?
Thank you for pointing us to these references. After reviewing these references, we have decided not to include them in our Discussion of our results because the methods are potentially too different from one another (e.g., unsupervised statistical learning of sequences vs. supervised learning of speech categories). We do not believe that we would be able to incorporate these references in the manuscript without providing substantial additional context and that is out of scope for the current article.

R1.C8: p. 8, lines 164--165: The phrasing is a bit awkward here.
We have updated this to: "The ability to accurately identify novel exemplars is a hallmark of categorization" (page 8, line 166-167)

R1.C9: p. 12, line 247: "were produced" should be "was produced".
We have updated this to "was produced"

R1.C10: p. 14, lines 290--291: There are a few differences between the study you cite here in support of not using an accuracy filter, and your own research (e.g., in-lab student vs. online

sample) that make me wonder whether including an accurary threshold would be equally unnecessary in your case. I'd be interested to at least know how well participants performed in the arithmetic task (e.g., was the proportion of participants who did poorly similar to that in Đokic et al.'s sample?). Actually I see from looking at the supplemental materials that you do provide arithmetic task performance data; perhaps good to mention this here.
We now directly report average performance on the arithmetic task in the main text (page 14, lines 298-300) and point interested readers to the Supporting Information for more information.

R1.C11: p. 14, line 296: Did you consider allowing \delta_s (i.e., the offset parameter) to vary across participants as well (and assess its relationship with OSPAN scores)? It seems plausible to me that part of learning stimuli in a novel language is developing the ability to more rapidly encode them, so that the decision (/categorisation) process receives good information more rapidly.
We did allow the offset parameter to also vary across participants. We have now clarified this explicitly on page 15, lines 308-310: "The model lets the parameters $\mu_{d,s}$, $b_{d,s}$, and δ_s to vary between participants, which accommodates the substantial variability across participants."

R1.C12: p. 15, line 307: What priors did you assign to the parameters?
The full details of our drift-diffusion model, including the choice of the priors, the resulting posterior, associated computational machinery for model fitting and posterior inference, simulated and real data illustrations, etc. can be found in Paulon et al (2021). A full discussion of these details is outside of the scope of this article. Following your comments, however, we now present a non-technical summary of the model in the Supporting Information.

R1.C13: p. 16, lines 338--346: Would a logistic model (e.g., via glmer) not be a better choice than a linear one here, given accuracy is restricted between 0 and 1 (/100)?
Even though accuracy is restricted between 0 and 1, the mean *accuracy values are continuous and normally distributed and are therefore appropriate for a linear analysis. Because our focus is on fitting the model to the data rather than predicting out-of-sample values, we are not concerned about the model fitting values outside of the accuracy ranges.*

R1.C14: p. 17, lines 354--360: To enable the reader to better grasp the meaning of these effects, it might be useful to provide some information about the distribution of WM scores (e.g., to emphasise that a one-unit difference in WM scores is a fairly small increment); otherwise they may seem trivial.
Thank you for this suggestion. We have added more context to the text to remind readers that the OSPAN scores could range from 0 to 75. Page 19, line 397-401: "As a reminder, WM scores could range from 0 to 75, so even a relatively modest increase in WM score of 10 points would be associated with an additional increase in accuracy of 3.2% in the final block of session 1. A larger difference in WM score of 30 points would be associated with an additional increase in accuracy of 9.6% in this block."

R1.C15: p. 24, lines 490--491: When you say here that you "focus on the results for correct trials only", what exactly do you mean? Surely the model gives you parameter estimates that combine information from correct and error responses, no? In that case, how can you isolate the correct trials?

Please see detailed response to R1.C2a.

R1.C16: pp. 24--25, lines 509 on: Is there any reason for not just including WM capacity as a predictor in the diffusion modelling? Given the hierarchical nature of the diffusion model fitting, your individual participant parameter estimates are not independent, are they? Therefore the independence assumption of the regression analysis is violated.
Please see detailed response to R1.C2b.

R1.C17: p. 29, lines 620--625: The first thing that springs to my mind when I read this is Logan's instance theory of automatisation (e.g., Logan, 1988). If there is an initial stage of learning where a task is completed algorithmically, followed by expert responses resulting from LTM retrieval once enough instances are accumulated in memory, we would probably expect a fair bit of WM involvement in the first, but perhaps not so much in the second.
Thank you for pointing us to this reference. As we believe performance in this task is still below expert automated level of responding, we have decided not to include discussion of these results in the current article to keep the discussion in scope.

References:

Logan, G. D. (1988). Toward an instance theory of automatization. Psychological Review, 95(4), 492–527. https://doi.org/10.1037/0033-295X.95.4.492
Smalle, E. H. M., Muylle, M., Duyck, W., & Szmalec, A. (2021). Less is more: Depleting cognitive resources enhances language learning abilities in adults. Journal of Experimental Psychology: General, 150(12), 2423–2434. https://doi.org/10.1037/xge0001058
Smalle, E. H. M., Daikoku, T., Szmalec, A., Duyck, W., & Onen, R. M. (2022). Unlocking adults' implicit statistical learning by cognitive depletion. Proceedings of the National Academy of Sciences of the United States of America, 119(2), 1–9. https://doi.org/10.1073/pnas.2026011119
Unsworth, N., & Engle, R. W. (2007). The nature of individual differences in working memory capacity: Active maintenance in primary memory and controlled search from secondary memory. Psychological Review, 114(1), 104–132. https://doi.org/10.1037/0033-295X.114.1.104

Reviewer #2
The authors examined the influence of working memory on category learning performance. Unlike the other studies, which either only studied the effect of WMC on immediate category learning or the effect after the categories were fully learned, the authors examined the continuum between the categories that were first formed to the time when the categories were fully acquired. Thus, the authors bridged the gap between the past studies and provided new insight into category learning.

Overall, the experiment design was straightforward and logical. The flaws of doing an online study were carefully sidestepped and discussed. A large portion of chance-level participants might be dressed with a reward incentive. However, as a sufficient number of participants remained after excluding non-learners, running a new experiment might not be worth it. While the data is mostly sufficient to support the claim, the analysis is left to be desired.

There are two major concerns with the presented statistic:

R2.C1: 1. The blocks were treated as categories instead of time series. This stops the author from looking at the IV change across blocks and can only look at the difference between specific blocks. Most importantly, supporting the "stable" performance claim is difficult without looking at the performance across the blocks.
We thank the reviewer for this suggestion and appreciate the reviewer's concern (R2.C2) about the inflated alpha with this test as is. We have rerun the analyses with block as a continuous variable and reported the results.

R2.C2: 2. The statistic comes with vastly inflated alpha. The inflation might be the consequence of 1), as the interaction between the WMC and each block would have to be tested.
Please see response to R2.C1.

R2.C3: 3. The authors claim the effect of WM changes over sessions (P.29, 619). However, the only statistic associated with the claim was the gradually (but not wholly) increasing p-value which happened to become insignificant at the later session (again, but not all blocks). An actual test of WMC effect across sessions would be a direct test of this claim.
We now directly test the size of the WM effect on accuracy across sessions.

R2.C4: Drift diffusion model
The construction of the drift diffusion model also raised a question, why did the decision boundary depend on both the stimulus and the response? The authors used separate decision boundaries for four possible stimuli and four possible responses, i.e., 16 decision boundaries. Why? I can understand the difference in drift rate, as different stimuli would give different evidence for all possible responses. However, why would the decision boundaries change depending on the stimuli?
As the authors pointed out, the analysis only focused on the boundary leads to the correct response. Thus, implementing multiple boundaries based on the stimuli seems unnecessary and further explaination might be needed. If the extra boundaries were needed due to mathematical reasons. The authors needed to communicate it.
We have now clarified our choice to allow drift rate and boundary to vary across both stimulus and response on page 15, lines 312-326: "We allowed both drift rate and boundary to vary across both stimulus category and response and assume that participants gather evidence towards each of the four possible response options at different rates and different levels of response caution depending on the true identity of the stimulus category. The decisions participants make in this task are tied directly to the sound category. Exemplars from within a sound category share characteristics and differ from exemplars from other sound categories. Due to the stimulus characteristics, participants may accumulate evidence at different rates and with different criteria for the different stimulus-response combinations.

An example may help to further illustrate this point. Say that participants learned four categories that differed in pitch frequency (e.g., low pitch, medium-low, medium-high, and high). When a participant hears a sound that has a generally lower pitch, they should accumulate evidence faster and need less information to respond for low pitch or medium-low pitch categories relative to medium-high and high pitch categories. Because the stimuli vary in ways that cue category identity, we made the decision to fit separate drift rates and boundaries for each combination of stimulus and response category."

Furthermore, we also fit and compared the full model with a sub-model where the boundaries only depend on the response categories (but were still allowed to evolve with learning and vary between individuals). We compared the model fits using Watanabe-Akaike information criterion (WAIC), a popular approach for assessing the predictive performances of competing methods. WAIC is obtained by computing the log point-wise predictive density and then adding a correction reflecting the effective number of degrees of freedom to prevent from overfitting (see Gelman, Hwang, & Vehtari, 2014, for details). The original flexible model of Paulon et al. (2021) (which allows the decision threshold parameters to vary across both response and stimulus categories) produced significantly better WAIC values compared to the restrictive model (which allows the decision thresholds to only depend on the response categories). This indicates that, from a statistical model fitting point of view as well, the fully flexible model is vastly superior to the restrictive model in describing the data. We discuss these results on page 15-16 (lines 327-338) and present the results of this comparison in Table S5 in the Supporting Information.

Gelman A, Hwang J, Vehtari A. Understanding predictive information criteria for Bayesian models. Stat Comput. 2014;24: 997–1016. doi:10.1007/s11222-013-9416-2

Some minor points:

R2.C5: P.7, Table 1: I think it might be dangerous to call the drift rate the "effieciency" of evidence accumulation. A stronger signal would also lead to a larger drift rate and is different from the "efficiency".

We have removed reference of the drift rate as 'efficiency' and instead referring to the drift rate as rate of evidence accumulation.

R2.C6: P.7, Line 146: "A subset of these participants (107/195) return for the current study. This line doesn't fit into the previous sentences; maybe a misplacement?

*This sentence is intentionally placed and follows from reference to McHaney et al. in the prior sentence. We have made the verb here past tense to clarify this sentence on pages 7-8, lines 148-150: "among all **195** participants, WM was positively related to learning. A subset of these participants (107/195) return**ed** for the current study."*

R2.C7: P.10, Line 195: "The rate of evidence accumulation reflects the quality of information extracted from the stimulus, with faster rates reflecting a more efficient evidence accumulation process." This could also be on the increased efficiency of retrieving and comparing the examplers from memory. It would be difficult to identify whether the increase in drift rate was due to better information extraction or other categorization processes.

We have added this additional explanation to the text in the next sentence, page 10, lines 200-202: "The evidence accumulation process may also reflect efficiency of retrieval or access to categorization exemplars or other representations in memory."

R2.C8: P.10, 200: Why can't the participants with higher WM to have the same decision threshold and respond faster without sacrificing the accuracy?

We have added this additional explanation in the text, page 11, lines 218-220: "Alternatively, individuals with higher WM capacity may have sufficient resources to maintain similar decision thresholds as individuals with lower WM capacity, enabling them to respond faster without making sacrifices in performance."

Reviewer #3
The authors investigate the relationship between working memory (WM; as measured by Operation Span) and learning Mandarin tone categories that were novel to the participants using a longitudinal, multi-session design. The authors examined both early learning as well as later retention/learning with follow-up sessions scheduled one month after the first and two months after the second. The authors also looked at whether there were differences for participants who successfully learned the tone categories vs. those who did not. For the latter, it appears that non-learners are generally disengaged from the task and may be seeking to minimize time spent in the experiment.

The key findings were that WM was positively associated with early learning, replicating several previous findings. Results for knowledge retention across sessions, however, showed no link, suggesting that WM is mainly involved in early, rather than later stages of learning. This is possibly due to the requirement of having to consider many different hypotheses early on in learning vs. only having to refine a single hypothesis (or a smaller set of viable ones) after performance has stabilized.

The authors also employ diffusion modeling to examine links between quality of evidence (drift rates, or accumulation rates) and response caution (decision thresholds). For learners, there was evidence that higher WM individuals showed not only more efficient evidence accumulation, but also greater response caution.

I found the manuscript to be very well written and exceptionally clear. The study was well-motivated, and the conclusions generally followed from the results of the analyses. My overall disposition is quite positive. I do have a few comments, mostly minor, that I would like to be addressed in a revision. I detail these below.

Major Comments

R3.C1: 1 - Feedback was only provided as correct/error, but with a 4-category structure, this is not especially informative. Better to provide category feedback (i.e., information about response accuracy along with what the correct category was). This should be acknowledged as a limitation, as it may have contributed to poor performance leading to task disengagement. Because error feedback is highly ambiguous, it is that much more difficult to learn effectively.
We have now acknowledged our use of correct/incorrect only feedback as a potential limitation to the study, making learning potentially more difficult than with full feedback. However, we also contextualize this with prior work that has demonstrated that Mandarin tone learning specifically is enhanced by minimal (correct/incorrect only) feedback relative to full feedback (e.g., "correct, that was category 1") (Chandrasekaran, Yi, & Maddox, 2014).

Chandrasekaran B, Yi H-G, Maddox WT. Dual-learning systems during speech category learning. Psychon B Rev. 2014;21: 488–95. doi:10.3758/s13423-013-0501-5

Page 38, lines 807-812: "Finally, participants learned four difficult categories with minimal feedback (e.g., "correct" or "incorrect"). Because this kind of feedback is ambiguous when the response is incorrect, it is possible that performance may have improved if we had provided full feedback (e.g., "correct, that was category 1"). However, prior work has demonstrated that

Mandarin tone learning, as we examined here, is better with minimal feedback relative to full feedback [89]. Future studies will need to address the role of WM in learning with full and minimal feedback."

R3.C2: 2 - The racing diffusion model used here is set up in a way that differs from typical applications. The current model is configured in a way that is extremely flexible, raising questions about the viability of simpler, potentially more theoretically plausible, configurations. More seriously, the model configuration raises falsifiability concerns about the authors' application of the model (see Jones & Dzhafarov, 2014).

My main concern is with the way decision thresholds are set. Based on my reading of the description on page 14, it seems that decision thresholds for the four categories are conditioned on the category of the stimulus (i.e., if a Category 1 stimulus is presented, the thresholds for the categories might be set to A, B, C, and D, but if a Category 2 stimulus is presented, they might be set at E, F, G, and H). If this is correct—apologies if I have misunderstood—the assumption is psychologically implausible, at it allows the system to adaptively configure itself in response to the stimulus category, but prior to identifying the stimulus category. A plausible configuration could still allow thresholds to differ across categories, but independent of the current stimulus.
Jones, M., & Dzhafarov, E. N. (2014). Unfalsifiability and mutual translatability of major modeling schemes for choice reaction time. Psychological Review, 121(1), 1–32. https://doi.org/10.1037/a0034190
Please see our detailed response to R2.C4.

R3.C3: 3 - Related to this is whether a simpler model might provide a satisfactory account of the data. Given the unfamiliarity of the categories to the learners, is there an a priori reason to assume differences in decision thresholds? How well does a model with a single threshold common across all categories (and all stimuli) perform? The threshold(s) could still vary with learning, but testing a common threshold model against one that allows category-specific thresholds to vary would be prudent.
In addition to our detailed response to R2.C4, we do not think a shared decision threshold (across all response and stimulus categories) is appropriate for our problem. To our knowledge, in most conventional drift-diffusion models for binary choices, a parameter is included to model possible inherent bias toward or against one of the two response categories, so the boundaries are in fact not at equal distances from the starting point of the underlying diffusion processes. Race models like ours, having different thresholds for different response (and stimulus) categories, naturally do not need such additional bias parameters.

Minor Comments
R3.C4: 4 – In Table 1, "enhanced learning and motivation" is listed as the hypothesized role of WM in later learning. Should this be "maintenance of knowledge"?
We have included "enhanced attention and motivation" as a hypothesized role of working memory in later learning as it relates to general accuracy in sessions 2 and 3. This is an operationalization of 'later learning.' In contrast, maintenance of category knowledge is reflected in the difference in accuracy between the final block of a session and the first block of the next session. As such, maintenance of knowledge and later learning have their own rows in Table 1.

R3.C5: 5 - Line 53 – Should "temporal" be "temporary"?
We have updated this to "temporary"

R3.C6: 6 - Line 129 – The description of the two analyses considered could be explained more clearly in the parenthetical comment (i.e., learners only vs. all participants, including both learners and non-learners).
*We included the parenthetical not to explain the analyses, but to operationally define learners and non-learners. We have changed the text to reduce confusion on page 6, lines 130-132: "*We examine both the entire set of participants and participants who perform at above-chance levels (i.e., learners vs. non-learners who do not perform at above-chance levels)."

R3.C7: 7 - Is the "non-decision time" offset, δs, allowed to vary across participants (and potentially learning)? If not, is there a reason why it is fixed across individuals/blocks?
Please see detailed response to R1.C11.

R3.C8: 8 - There have recently been a number of models that combine learning assumptions with diffusion decision mechanisms (e.g., Fontanesi et al., 2019, Miletic et al., 2021; Pedersen et al., 2017; Sewell et al., 2019). Do the authors think that their conclusions would be any different if one of these other models were used instead?
Fontanesi, L., Gluth, S., Spektor, M.S. et al. A reinforcement learning diffusion decision model for value-based decisions. Psychon Bull Rev 26, 1099–1121 (2019). https://doi.org/10.3758/s13423-018-1554-2
Steven Miletić, Russell J Boag, Anne C Trutti, Niek Stevenson, Birte U Forstmann, Andrew Heathcote (2021) A new model of decision processing in instrumental learning tasks eLife 10:e63055. https://doi.org/10.7554/eLife.63055
Pedersen, M.L., Frank, M.J. & Biele, G. The drift diffusion model as the choice rule in reinforcement learning. Psychon Bull Rev 24, 1234–1251 (2017). https://doi.org/10.3758/s13423-016-1199-y
Sewell, D.K., Jach, H.K., Boag, R.J. et al. Combining error-driven models of associative learning with evidence accumulation models of decision-making. Psychon Bull Rev 26, 868–893 (2019). https://doi.org/10.3758/s13423-019-01570-4
The drift-diffusion mixed model we used here is specifically tailored for multi-category learning by participants over several blocks of training trials. The models mentioned above are all interesting works. However, these models are not clearly applicable to this multi-category learning problem over blocks of trials. These models are designed to examine fixed effects only, are designed for binary outcome scenarios and include different contexts (e.g., reinforcement learning problems with specific reward structures: Pedersen et al., 2017; Fontanesi et al., 2019).

Below, we comment further on the key differences between our models and several models mentioned by the reviewer to highlight the difficulty in comparing across models here: Sewell et al. (2019) examined a drift-diffusion model with exponentially evolving as well as a freely varying block-wise dynamic model for the drifts. They also introduced an integrated model for error-driven associative learning. However, these models were designed to examine behavior in a probabilistic learning task in a binary response category setting with no random effects. The race model of Miletic et al., (2021) accommodates multi-category responses but is restricted to

static fixed effects setting. As such, these models are therefore not directly applicable to our complex multi-category multi-subject multi-training-block mixed effects longitudinal experimental design. However, since the basic foundation of all these models is drift-diffusion based evidence accumulation, we do not think that their potential adaptations for our setting will produce starkly different results. A full comparison across different models in this setting is outside of the scope of the current article.

R3.C9: 9 - Were fast responses filtered out of the data? Responses faster than 150-200 ms are usually regarded as anticipatory rather than stimulus-driven.
*Yes, very fast and very slow responses were excluded from analyses by removing the top and bottom 1% of data based on reaction times. We have better clarified the data inclusion in the Methods section, page 16, lines 339-340: "*The data were filtered to exclude very fast and very slow responses by removing the top and bottom 1% of trials based on reaction time."

R3.C10: 10 - Page 26 – The interpretation of decision thresholds in non-learners should be phrased more cautiously. Since most of the WM correlations are non-significant, there are concerns about false positives. Given that only one of the correlations outside of Session 1 was significant, I think the authors should be clear that the evidence that non-learners with higher WM have lower decision thresholds is relatively weak/inconsistent. That said, the more systematic trend in Session 1 is perhaps indicative of general task disengagement (or optimizing performance to minimize time in the experiment). Why this would only manifest in Session 1 is unclear, however, hence my general view that these results should be interpreted with greater caution.
We have removed these analyses entirely from the current version of the manuscript as we focus on the effect of WM on our different measures for learners only. As a result, this point no longer applies.

R3.C11: The idea of non-learners trying to minimize time in the experiment is related to previous discussion along these lines by Hawkins, Brown, Steyvers, & Wagenmakers (2012). Hawkins, G.E., Brown, S.D., Steyvers, M. et al. An optimal adjustment procedure to minimize experiment time in decisions with multiple alternatives. Psychon Bull Rev 19, 339–348 (2012). https://doi.org/10.3758/s13423-012-0216-z
Thank you for pointing us to this paper, we have now cited this in the Discussion (page 33, lines 696-698).

R3.C12: 11 - In the abstract, it is mentioned that WM is positively associated with generalization, but this doesn't seem to be the case given the analyses reported on pp. 22-23.
We have updated the abstract to better reflect the results.
