# Supplementary material for: Individual differences in working memory impact the trajectory of non-native speech category learning
Source: PLoS One. 2024 Jun 10;19(6):e0297917. doi: 10.1371/journal.pone.0297917 (PMC11164376; doi:10.1371/journal.pone.0297917)
Supplement: Attachment — Submitted filename: 2023-11-15_ResponsetoReviewers.docx [file pone.0297917.s003.docx]

*We thank the editor and three reviewers for consideration of our revised manuscript. Below, we briefly summarize the additional changes we have made to the manuscript in response to reviewer comments. We then respond to each point raised by the reviewers with our responses in blue italics.*

***Summary:***

- Utilization of a more constrained version of the model (which allows drift rates to vary across stimulus and response categories, but boundaries only vary across response categories). Further, we arrived at this model by selecting among different versions of a more constrained model, which are now further detailed in the Supporting Information.
- Include more information about model fit in the Supporting Information.
- Minor changes for readability and simplifying the Results section.

**Editor**

Thank you for submitting your manuscript to PLOS ONE. I have sent your paper back to the original three reviewers of your prior submission. All reviewers agree that your paper is substantially improved. Yet, two of the reviewers feel that you have not addressed their concerns enough about the plausability of the model specified. This is a relatively big concern because model fit alone cannot be a basis to decide which model to use - when a model has several parameters, reasoning about the underlying model structure and meaningful implementation also needs to be used to constrain the model. Here, both reviewers feel that it makes no sense to allow response boundary to vary with the stimulus category. The reasoning for this is standard in the literature. Hence I agree with Reviewer 1 that you need to use a more constrained version, even if it slightly misfits the data. On that note, it would be important to present evidence of model fit. I think this should be presented as supplementary materials to not make the paper too long and cumbersome to read.

*In direct response to comments from the Editor as well as Reviewers 1 and 3, we have used a more constrained version of the model, which does not allow boundaries to vary across different stimulus categories. Additionally, we have added more evidence of model fit to the Supporting Information.*

*We would also like to reiterate that the version of the model that allows both drift rates and boundaries to vary with the stimulus category is relatively novel to the field and is the basis of an NSF award to Dr. Sarkar and Dr. Chandrasekaran, but it does provide significantly better fit to the data. We understand that there is concern about the psychological plausibility of this version of the model (hence we have used a more constrained version here). While not fully elaborated therein, we can assure (three of the original authors are co-authors here as well) that the original decision to allow this flexibility in Paulon et al. (2021) was well-thought-out one, with significant deliberation regarding the biological plausibility, and not a mere oversight.*

*Since the psychological plausibility of this model is outside of the scope of the current article, we plan to address this question in a separate future work. Our general rationale for the original model is as follows. (1) If we can assume (as is typical in conventional race models) that when an input stimulus category is presented, only the drift-diffusion processes corresponding to that specific input category (via drift rates varying by stimulus category) come into play in accumulating evidence in favor of different alternatives (while the other accumulators corresponding to other stimulus categories remain completely absent), we should be open to allowing the input category information influence other aspects of the underlying processes (including the boundaries) as well. (2) The most important timepoint to consider while interpreting the decision boundaries is at the time of decision, not at the beginning of or during the evidence accumulation process. Allowing the boundaries to be flexible based on incoming information from the stimulus reflects the ability to dynamically adjust behavior based on stimulus information. This ability may not be used in tasks and approaches that commonly use DDMs (e.g., detection, go-no go), but is arguably very important for perceptual categorization. (3) There is strong statistical evidence in favor of the fully flexible model (drift rates and boundaries allowed to vary over time and across responses and stimulus categories). If a reasonable argument for psychological plausibility can be made (see point 2 above), then we should take seriously the better statistical fit of this fully flexible model.*

*Here (for the purpose of the response to reviewers only) we provide an assessment of model fit for four different models which only differ in terms of how they treat the boundaries. They are*

1. *flexible: the original model of Paulon, et al. (2021) that allows the boundaries* $b_{d,s}^{(i)}(t)$ *to vary with both the stimulus* $s$ *and the response* $d$ *as well as with time* $t$*;*
2. *fixed: a sub-model that allows the boundaries* $b_{d}^{(i)}(t)$ *to vary with the response* $d$ *as and with time* $t$ *but not with the stimulus* $s$*;*
3. *constant: a sub-model that allows the boundaries* $b_{d,s}^{(i)}(t)$ *to vary with both the stimulus* $s$ *and the response* $d$ *but not with time* $t$*;*
4. *fixed-constant: a sub-model that allows the boundaries* $b_{d}^{(i)}$ *to vary with the response* $d$ *but not with stimulus* $s$*, nor with time* $t$*.*

*All models accommodate subject heterogeneity by allowing the boundaries to vary with the subject index* $i$*. As anticipated, the original ‘flexible’ model provides the best model fit, followed by ‘fixed’, then ‘constant’, and finally the most restrictive ‘fixed-constant’.*

***Table R1***

*Model fit assessed by -2*WAIC for the original model of Paulon et al. (2021) and three different sub-models. The smaller the reported model assessment value, the better.*

|  | *Session 1* | *Session 2* | *Session 3* |
| --- | --- | --- | --- |
| *Model with* $b_{d,s}^{(i)}(t)$ *(flexible)* | *92691.86* | *82792.24* | *76261.58* |
| *Sub-Model with* $b_{d}^{(i)}(t)$ *(fixed)* | *93409.11* | *83795.52* | *77647.32* |
| *Sub-Model with* $b_{d,s}^{(i)}$ *(constant)* | *95203.27* | *84444.15* | *78203.95* |
| *Sub-Model with* $b_{d}^{(i)}$*(fixed-constant)* | *95697.74* | *85671.44* | *79635.96* |

*Nevertheless, in the end, in the revised version of the current article and the Supporting Information document, we have decided to follow Reviewer 1’s first suggestion (see R1.C1 below) and used the more constrained ‘fixed’ version of the model, which does not allow boundaries to vary across different stimulus categories. We have edited the manuscript accordingly and the results for decision threshold are changed from previous versions. The results for the evidence accumulation rate are nearly identical to previous versions. The results overall have thus not changed substantially and the major conclusions very much remain the same. We hope this comprehensive revision that aligns with the reviewer perspectives addresses the concerns.*

**Reviewer #1**

**R1.C1:** The authors have effectively responded to most of my concerns, and I think the revised manuscript is an improvement on its predecessor in terms of its accessibility and readability. That said, I still have serious concerns regarding the psychological plausibility of a model of this type in which decision thresholds depend on knowledge of the stimuli being categorized, and the authors' response on this point has not convinced me otherwise. (I also read the paper by Paulon et al. very carefully to see whether I was missing something that had been described there, but did not come across any such thing.) A model that is psychologically implausible is problematic, irrespective of how well it might fit the data, because it is unclear how its parameters can be interpreted.

It is possible that people's decision boundaries really do change in a sort of cascaded manner, with processed low-level stimulus information feeding forward into the decision processes used for higher-level categorization. However, as far as I can see this would be an entirely different model to the one applied here.

I don't want to be totally un-constructive with my comments, so let me suggest three potential paths forward on this point. First, the authors could focus on the more restricted (but psychologically plausible) model instead, and revise the paper accordingly. Second, the authors could retain the current model, explicitly discuss its psychological implausibility, and explain in detail what sort of underlying processes might lead such a model to provide a better fit than the more restricted but theoretically sounder alternative. Third, the authors could do a bit of both: Cover the results from the restricted model AND the results from the flexible one, and discuss what it is that allows this model to fit the data better despite it being implausible, and what this might imply for the underlying processes. (Of course, I leave the possibility open that there are additional paths forward that I have neglected.)

*In direct response to this comment (as well as similar comments made by Reviewer 3 and the Editor), we have used a more constrained version of the model, which does not allow boundaries to vary across different stimulus categories (Reviewer 1’s first suggestion here). Please see our detailed response to the Editor above for additional information.*

In addition to this, I have just a few very minor, language-related comments:

**R1.C2:** p. 29, lines 610--612: This sentence is a little confusing. Do you mean that the increase across blocks was not significantly different from session 1? Or the rate itself?

*Based on overall edits to the results, this sentence is no longer in the manuscript.*

**R1.C3:** p. 29, lines 619--620: "changed across blocks, sessions"---a word missing here?

*Based on overall edits to the results, this sentence is no longer in the manuscript.*

**R1.C4:** p. 30, lines 634--635: I suggest changing "associated with an additional decrease in threshold" to "associated with a relative decrease in threshold" (or similar), since the baseline relationship for the session was positive.

*Based on overall edits to the results, this sentence is no longer in the manuscript.*

**R1.C5:** p. 34, line 720: Who is the "them" in this sentence?

*We have changed this to clarify:* “helps **learners**”

**Reviewer #2**

**R2.C1:** After the revision, the authors addressed all my previous concerns. Hence, I recommend accepting the manuscript.

The only minor gripe is that the statistic results became too long and cumbersome to read. Moving part of the results to the supplementary material might be better for readability. However, I would be okay with the paper staying the same.

*Based on overall edits to the results to accommodate the more constrained model, the results are much shorter. We believe that these changes enhance readability.*

**Reviewer #3**

The authors have addressed most of my comments from their initial submission. However, there remain two outstanding issues that have not been adequately addressed. These map onto my 2nd and 3rd points (R3.C2 and R3.C3, respectively) raised in my initial review.

**R3.C1:** Regarding R3.C2, I still have serious concerns about the decision to allow both drift rates and decision thresholds to vary as a function of stimulus-response combination. For drift rates, this is perfectly fine, and in keeping with conventional practice with regards to fitting data. For decision thresholds, however, this level of flexibility introduces a theoretical circularity that renders the model psychologically implausible.

It is fine for decision thresholds to vary as a function of response—participants may be more or less cautious about making particular responses. Indeed, this is how racing accumulator models (such as the racing diffusion model the authors use here) address response bias issues. Where things become problematic is when decision threshold is allowed to vary as a function of the stimulus. The core objection is this: If the outcome of the decision process is to identify the stimulus as belonging to a particular category, the system cannot be configured in a stimulus-specific manner because this requires foreknowledge of the outcome of the categorization process. Put another way, conditioning the threshold on the identity of the stimulus requires the system to have already categorized the stimulus for the purposes of threshold setting. In which case, there is no need for a subsequent evidence accumulation stage because the stimulus has already been categorized implicitly.

I appreciate that the authors have presented statistical support for allowing threshold to vary according to the stimulus, but this does not address the theoretical/psychological objection to configuring the model in this way. That is, the WAIC comparisons deal with a quality of fit issue, but they do not deal with the more substantive theoretical interpretation of the model.

This raises a point I had overlooked in my original review—there is no visualization of model fit. Is it the case that good fits can only be achieved by allowing the model to estimate thresholds in a stimulus-dependent way? Plotting either quantile-averaged data against quantile-averaged model predictions would be one way to check the correspondence between theory and data. Another way—perhaps more appropriate given the large sample nature of this study—would be to generate scatterplots showing predicted vs. observed RT quantiles for correct responses and errors as well as choice probabilities.

*In direct response to the first point in this comment (as well as similar comments made by Reviewer 1 and the Editor), we have used a more constrained version of the model, which does not allow boundaries to vary across different stimulus categories (Reviewer 1’s first suggestion here). Please see our detailed response to the Editor above for additional information.*

*To the second point regarding the visualization of model fit, we have now plotted the predicted versus observed reaction times (and responses) to the Supporting Information (Figure S3).*

**R3.C2:** The other issue that I still think needs addressing, regarding R3.C3, extends on the above point. Are response-dependent thresholds required to achieve good fits to data? The authors correctly note in their original response that an accumulator framework does not require a common threshold setting for each accumulator, but my question was whether a common threshold value suffices to explain the data. It may well be the case that participants have response biases that lead them to be more reluctant/cautious about making some category responses over others, but demonstrating that this flexibility is needed is important for ensuring explanatory parsimony in terms of the preferred model.

*Please see our detailed response to the Editor above.*
