# Supplementary material for: Individual differences in working memory impact the trajectory of non-native speech category learning
Source: PLoS One. 2024 Jun 10;19(6):e0297917. doi: 10.1371/journal.pone.0297917 (PMC11164376; doi:10.1371/journal.pone.0297917)
Supplement: Attachment — Submitted filename: 2024-01-11_ResponsetoReviewers.docx [file pone.0297917.s004.docx]

*We thank the editor and reviewers for consideration of our revised manuscript. We respond to each point raised by the reviewers with our responses in blue italics.*

**Reviewer #1**

The authors' current version of the manuscript has successfully addressed all the major concerns I previously had. There are only a few minor language issues I noticed while re-reading, which I detail below:

**R1.C1:** p. 6: At the top of the page, the reference to the McHaney et al. paper is numbered as 10, but should be 12.

*We have fixed this.*

**R1.C2:** p. 8: "We will assess generalization in each session by presenting learners with novel stimuli spoken by novel talkers that they do not encounter during training and never receive feedback about the correct category." This seems ungrammatical at the end. Maybe "…that they do not encounter during training, without providing feedback about the correct category" would be better?

*We have made the suggested change.*

**R1.C3:** p. 11: "…without making sacrifices in performance" should perhaps be "without making sacrifices in accuracy" (since response time is also part of performance).

*We have made the suggested change.*

**R1.C4:** p. 11: The reference to the McHaney et al. paper is numbered wrongly again.
*We have fixed this.*

**R1.C5:** p. 15: Line 319 starts with "Click or tap here to enter text."

*We have fixed this.*

**R1.C6:** In addition, I thank the authors for attempting to explain the validity of their original model. I'm genuinely interested in how this would work, and though I don't really understand the argument made in the response letter, I'll look forward to seeing their future work on this topic. To that end, if I were a hypothetical reviewer of that work, one thing I'd definitely want to see would be model recovery simulations that show that the more flexible model is selectively preferred when data are generated by a model with boundaries that change within a trial (but not when they are generated by a model without such a feature).

*We thank the reviewer for their comments – this will surely help us in preparing this future work for publication.*

**Reviewer #3**

The authors have addressed my comments and concerns in their revision. I have one small suggestion for a further analysis of drift rates that the authors might wish to consider (though I don’t think it is essential). It may provide some further insight into the extent to which WM relates to extraction of evidence in general vs. more focused extraction of categorically diagnostic information.

**R3.C1:** 1 - I understand the focus on drift rates for the correct response option (i.e., when stimulus and response match). This indexes absolute evidence accumulation for the one option. It would be interesting to know whether there are discriminability differences as well, however. If correct drift rates were normalized across the sum across accumulators, you could further consider discriminability of the response options. This is potentially important because it pertains to whether higher WM is related to better extraction of evidence overall vs. extraction of diagnostic evidence that differentiates the categories.

*We appreciate this comment but see this analysis as outside of the scope of the current investigation. We have made a note about pursuing this possibility in future work.*

**R3.C2:** Line 319 – Editing issue, “Click or tap here to enter text”.

*We have fixed this.*

**R3.C3:** Lines 319-320 – Was RT filtering based on slowest/fastest 1% of responses in the entire data set, or was filtering done separately for each participant?

*We have clarified this (line 323-324, page 15):* The data were filtered to exclude very fast and very slow responses by removing the top and bottom 1% of **all** trials **across** **all** **participants** based on reaction time.
